# The roles and training of primary care doctors: China, India, Brazil and South Africa

**DOI:** 10.1186/s12960-015-0090-7

**Published:** 2015-12-04

**Authors:** Robert Mash, Magda Almeida, William C. W. Wong, Raman Kumar, Klaus B. von Pressentin

**Affiliations:** Division of Family Medicine and Primary Care, Stellenbosch University, Cape Town, South Africa; Division of Family Medicine and Primary Care, University of Fortaleza, Fortaleza, Brazil; Department of Public Health, Federal University of Ceará, Fortaleza, Brazil; Department of Family Medicine and Primary Care, The University of Hong Kong, Pokfulam, Hong Kong; (Family Medicine) Academy of Family Physicians of India, New Delhi, India

**Keywords:** Primary care physicians, General practitioners, Family physicians, Primary health care, Universal coverage, Physician’s role, Graduate education, China, India, South Africa, Brazil

## Abstract

China, India, Brazil and South Africa contain 40% of the global population and are key emerging economies. All these countries have a policy commitment to universal health coverage with an emphasis on primary health care. The primary care doctor is a key part of the health workforce, and this article, which is based on two workshops at the 2014 Towards Unity For Health Conference in Fortaleza, Brazil, compares and reflects on the roles and training of primary care doctors in these four countries.

Key themes to emerge were the need for the primary care doctor to function in support of a primary care team that provides community-orientated and first-contact care. This necessitates task-shifting and an openness to adapt one’s role in line with the needs of the team and community. Beyond clinical competence, the primary care doctor may need to be a change agent, critical thinker, capability builder, collaborator and community advocate. Postgraduate training is important as well as up-skilling the existing workforce. There is a tension between training doctors to be community-orientated versus filling the procedural skills gaps at the facility level. In training, there is a need to plan postgraduate education at scale and reform the system to provide suitable incentives for doctors to choose this as a career path. Exposure should start at the undergraduate level. Learning outcomes should be socially accountable to the needs of the country and local communities, and graduates should be person-centred comprehensive generalists.

## Background

The World Health Report (WHR) of 2008 reminded us that primary health care (PHC) should still be the foundation of effective health systems [1]. PHC is also the best approach to achieving universal health coverage [2] and a fundamental requirement for achieving the Sustainable Development Goals [3]. Unfortunately, since the concept of PHC was created at Alma Ata in 1978, many countries have failed to establish effective PHC [1]. PHC is often neglected; diluted into poor quality health care by inadequately resourced, trained and scarce health workers; or reduced to a series of selected activities and vertical disease-orientated programmes [1]. The WHR recommended a number of reforms that are required to establish more effective PHC: improving health equity by focusing on universal coverage, making health systems more people-centred by changing the focus of service delivery, making governance of the system more reliable by developing leadership, and being more community-orientated with a public health perspective [1]. The WHR also noted that successful PHC systems usually involve a primary care doctor with postgraduate training in family medicine or general practice. The aim of this article is to explore the roles and training of the primary care doctor in four countries that are all committed to improving their PHC systems.

The material in the article was presented at two workshops of The Network: Towards Unity For Health Conference in Fortaleza, Brazil, in 2014 [4]. A primary care doctor with a background in academic family medicine was identified in China, India, Brazil and South Africa. Each doctor produced a poster on the roles of primary care doctors and another on the approach to training in their country. These posters were presented at the workshops and discussed by an international group of delegates attending the conference. After the workshop, each presenter wrote up their presentation in a standardised template. This article integrates and compares this information and reflects on the lessons learnt.

## Main text

### Country profiles

The four countries compared in this article represent 2.85 billion people, which is around 40% of the world’s population (Table 1) [5]. They have also been seen as emerging economies and are key members of the BRICS (Brazil, Russia, India, China, South Africa) group [6]. Delivering effective PHC within these four countries would therefore have a major impact on global health and development.

**Table 1:** **Country comparison for population and health care expenditure** **[** 5 **]**

**Country**	**Population (millions)**	**% rural population**	**Number of provinces or states**	**% population using private health care**	**% GDP on health**	**% GDP spent in public sector**
South Africa	53	36	9	16	8.9	4.3
China	1357	47	34	8	5.6	3.1
Brazil	200	15	22	25	9.7	4.7
India	1252	68	29	71	3.8	1.3

*GDP* gross domestic product.

All these countries face problems of inequity between urban and rural areas, geographic regions and social classes. In South Africa, which has one of the world’s highest Gini coefficients, inequality is still largely defined along racial lines as a legacy of apartheid [7]. Brazil spends the largest percentage of gross domestic product on health, while India has the poorest health care financing at only 3.8% [5]. In South Africa and India, the spending is also significantly skewed towards expenditure in the private sector for only a small part of the population.

### Current policy position on universal coverage and commitment to PHC

These countries have all made a commitment to universal health coverage through PHC. In India, the debate on universal health coverage continues, despite a policy document being published in 2011, although some states have experimented with health insurance [8]. The National Health Assurance Mission is talking of insurance to cover a list of medication, diagnostic services and services at the secondary/tertiary level in public-private partnerships [9]. In South Africa, the commitment to PHC was made in 1994, with the fall of the apartheid system, but a policy commitment to develop a national health insurance scheme was only made in 2011 and is not yet implemented [10]. China passed a social insurance law in 2011, which has been implemented for both urban and rural populations [11]. Brazil made a much earlier commitment to universal health coverage in 1988 and to PHC in 1991, and its unified health system today is a combination of both public and private health insurance [12].

All countries have recently been implementing strategies to strengthen PHC in order to prepare for or improve universal coverage. For example, in India, the National Rural Health Mission has upgraded 8250 primary health care facilities and 2313 facilities for first referral [13], while in South Africa, an Ideal Clinic policy is attempting to align all primary care facilities with a set of national standards [14]. China has also been establishing a primary medical care service infrastructure composed mainly of rural township centres, village clinics and urban community health centres (CHCs), to strive for equal access to basic public health services for both urban and rural residents. China has also recently established a national essential medicines system and committed itself to improving PHC and public health services as well as access to public hospitals. In Brazil, the Family Health Strategy has been a cornerstone of strengthening universal coverage through community-orientated primary care teams, and coverage has risen from 6.4% of the population in 1998 to 61.6% in 2014 [15].

Governance of primary care is a key issue. In China, governance of primary care appears fragmented between a plethora of different state bodies, which results in fragmented implementation of policy and, for example, differing levels of coverage and access to health care by different insurance schemes. In South Africa, India and Brazil, governance is also largely decentralised to the province, state and municipal levels, respectively. In all these countries, decentralised governance comes at a price of variability in implementation of national policy, human and infrastructure resources and health care provision.

The financial issues from the patient’s perspective also vary. In South Africa, primary care is free in the public sector for the population below a certain income, while in Brazil, all public health facilities are free of charge for the whole population. In China, insurance coverage leaves gaps that require out-of-pocket expenses and hospitals are still required to be financially self-sufficient, which results in a perverse incentive to perform expensive investigations, procedures and treatments. Patients are suspicious and may perceive doctors as “hungry money grabbers”. Indeed, China has a high rate of assaults on doctors who are not perceived to be people-centred. In India, health care is largely privatised and most people have to pay out of pocket for their health care needs. The large metropolitan cities have a concentration of high-tech and expensive tertiary care private facilities, and this pattern is spreading to smaller cities and towns. Tertiary care public health facilities are often overburdened and short of resources due to the weak primary care system and absent referral system. Assault on physicians due to dissatisfaction is also a common occurrence in India.

### Current delivery system for PHC

All countries have a variety of primary care facilities in the public sector referred to by different names such as basic health unit (Brazil), community health centres and clinics (South Africa), village health posts (China) and primary care centres (India) as shown in Table 2. The organisation of services, scope of practice and mix of health professionals in these primary care facilities differ considerably.

**Table 2:** **Types of health care facilities at different levels of the health systems**

	**China**	**India**	**South Africa**	**Brazil**
Community level (first contact)	–	Accredited Social Health Activist	Ward-based outreach teams	Family health care teams and Basic Health Units
Primary level (first contact)	Village health posts, rural township centres, village clinics and urban community health care centres (CHCs)	Sub-centres and primary health centres	Clinics or community health centres	Basic health units and emergency units
District level (generalist hospital care)	Level 1 hospitals (care offered by specialists)	Community health centres and sub-divisional hospitals	District hospitals	District hospitals
Secondary and tertiary level (specialist hospital care)	Level 2 and 3 hospitals	District hospitals and medical college hospitals	Regional, tertiary and central hospitals	Regional, tertiary and medical college hospitals

In Brazil, the discourse is largely about community-based primary care with multi-professional family health care teams that include a doctor, nurse, nurse assistant and four to six community health workers [16]. The team is responsible for providing primary care to 3–4000 people in a defined geographic area, and each community health worker looks after 750 people in the community. The team also operates out of the basic health unit to provide comprehensive primary care and could also incorporate oral health teams (dentists and dental technicians).

In South Africa, the emphasis is on nurse-driven primary care services that are facility-based. Comprehensive care is offered, although certain services such as human immunodeficiency virus (HIV) and tuberculosis (TB) are run through vertical programmes. Nurses might be supported by visiting doctors on a weekly basis, and in larger community health centres, there would be a broader multi-professional team consisting typically of nurses, doctor(s), pharmacist, allied health professionals and lay counsellors. There is currently a policy to introduce ward-based outreach teams which would consist of community health workers and a nurse, supported by a doctor, looking after a designated group of households in the community (similar to the Brazilian model). This policy, however, is still in the early days of implementation [17].

In China, primary care is offered by general practitioners (GPs), including traditional Chinese herbalists, as well as nurses. They provide selected primary care services for common ailments, chronic diseases and preventive care such as vaccinations. However, some facilities, converted from township hospitals, might include a broader range of services. Unfortunately, the range of services and the types of medication provided are very limited, and they are perceived by the general public as poorly equipped and of low quality [18]. A culture of over-investigation driven by financial incentives has led to a belief that good care should be high tech and also a suspicion regarding the motivation of doctors. GPs lack clinical skills in a system that is more public health-orientated and not person-centred. It appears that the population is not supportive of community health workers after the scrapping of “barefoot doctors” in the 1950s. Many doctors have been re-directed from working as hospital specialists to working in primary care by the government and need appropriate re-training for their new role.

In India, primary care is offered by a wide variety of practitioners. PHC facilities offer both curative and public health programmes, with a strong focus on maternal and child health. The training and induction of 900,000 ASHAs (Accredited Social Health Activists) is considered one of the success stories and a key component towards strengthening of community-based primary care [19]. Although there is increased focus and visible improvement in selected programmes, the generalist curative services are less than satisfactory. Within the public health sector, you may have choices between allopathic doctors, AYUSH (Ayurveda, Yoga, Unani, Siddha, Homeopathy) practitioners, nurses and midwives, community health workers and ASHAs. Outside of the public sector, you can also consult registered medical practitioners (who actually have little formal training and are unlicensed), as well as traditional healers.

Most countries have a parallel system of private general practice. In South Africa, for example, the majority of general practitioners are in the private sector where they offer primary care to a small part of the population with insurance or occasional out-of-pocket payments. Their role is often more curative and business-orientated, and the challenge is to integrate a large proportion of these doctors into the national health insurance scheme. It is envisaged that a small private sector will continue to offer top-up care to those that can afford double insurance. In this situation, immediate access to a specialist may be a feature. A similar situation is seen in India, although private general practice is on the decline due to a lack of academic recognition and any uniform financial reimbursement system. Senior general practitioners continue working in their 60s and 70s or are on the verge of retirement. The newly qualified medical graduates are not opting for general practice as a vocation. In Brazil, there are few general practitioners in the private sector, and they are mostly linked to home care services. Points of care offered by health insurance plans are mainly specialised medicine, where the client has direct access to specialist care. In China, private primary care practice has only recently been allowed and is still in its infancy.

In Brazil and South Africa, the public sector primary care system is meant to have a gatekeeping function in that patients can only access secondary and tertiary health care by referral from primary care. In many parts of both countries, however, patients still bypass primary care and present directly to hospital level care due to a lack of trust in the quality of care. Those with private insurance often have direct access to specialist care, although managed care schemes are introducing a gatekeeping role for primary care. In China, there has been a tradition of direct access to hospital-centred, specialist and high-tech care, which the new PHC system and social insurance schemes are hoping to interrupt. At most of the health facilities in India, patients can have access to specialist care without any referral. The situation is more of a necessity rather than a choice due to a weak primary care system. The public tertiary care facilities, however, are often overcrowded and short of resources.

### Roles of the primary care doctor

As can be seen from the way PHC is delivered in these countries, the role of the primary care doctor may differ considerably.

In Brazil, each of the 47,028 family health care teams is required to have a doctor working in the basic health unit and community at least 50% of their time. Currently, any doctor can take on this role and may not be appropriately or specifically trained for it. In the team, the doctor is expected to prepare and participate in group activities, to perform home visits and to provide ambulatory primary care. Many of the teams rely on foreign or Cuban doctors. There are only around 4000 doctors trained as family physicians (with postgraduate training) to work in this context and therefore a shortfall of 43,000 family physicians (FPs) [17].

In South Africa, doctors are often performing outreach to primary care clinics from local district hospitals, from community health centres or even from private general practice. They may see more complicated patients once a week that have been booked for them by the nurse. Where doctors are employed full-time, they are often inexperienced and required to be there as a result of internship or community service. A number of career medical officers and FPs (with postgraduate training) are also available, particularly in larger urban centres.

In China, due to the short history of primary care doctors, many senior doctors are either public health physicians or specialist-converted GPs who were given the task to set up CHCs. Often, CHCs are set up by and affiliated to the district-level hospitals, and the staff bonuses are related to the number of referrals made and, hence, the work created for the “mother-ship” hospital. As a result, the government and the hospital management have often pushed undesirable public health tasks, such as measuring the prevalence of HIV, onto these primary care doctors and nurses.

In India, the undergraduate qualification is supposed to produce the “basic doctor” to be employed in the public health system. However, most of the newly qualified doctors prefer to enter hospital-based specialities instead of primary health care. A large number of vacancies have been filled by AYUSH (non-allopathic alternative system) physicians. Although a role for postgraduate family medicine has been envisaged in policy, designated posts within the health system have yet to be created.

### Approach to training of primary care doctors

The number of medical schools, generalist doctors and FPs is compared in Table 3. Human resources, including primary care doctors, are usually maldistributed within countries with a bias towards urban areas and private practice. In India, the medical schools themselves are maldistributed leading to a shortage of doctors in the northern states.

**Table 3:** **Number of medical schools, outputs and generalist doctors** **[** 5 **,** 27 **–** 29 **]**

	**Number of medical schools (population in millions per medical school)**	**Outputs (new doctors/year)**	**Medical practitioners** ^**a**^ **/10,000 population**	**Family physicians/10,000 population**	**Nurses and midwives/10,000 population**
South Africa	9 (5.9)	1300	3.7	0.1	51
Brazil	242 (0.8)	21,395	19	0.2	76
India	398 (3.1)	52,305	7	-	17
China	980 (1.4)	192,344	14	1.2	51

^a^Not specialists.

#### Undergraduate training

In South Africa, curricula only introduced exposure to primary care in the late 1990s (last 15 years), when PHC was emphasised in post-apartheid government policy and there was an international move towards community-based education. After graduation, doctors now do a 2-year internship (which includes 3 months of primary care) and a 1-year community service (often in an underserved area with primary care). Following this, doctors are free to enter private general practice or continue as a medical officer with no further training.

Since primary care is a relatively new discipline in medical schools in China, there are very few GP departments in medical schools, and even where there are, they tend to be set up and run by public health specialists. Hence, the exposure to primary care at the undergraduate level is minimal, and the discipline has a very low status.

In Brazil, the medical undergraduate course takes at least 6 years. Exposure to primary care and to community-based education in the medical curricula was introduced in the last 13 years. In the last 2 years of the medical course, there are four 6-month clinical rotations through internal medicine, surgery, paediatrics/obstetrics/gynaecology and public health, of which the latter includes exposure to primary care. Since 2001, in many medical schools, the students are exposed to a more community-based curriculum from the first year.

In India, the undergraduate training consists of 5 years and 6 months, which includes 1 year of compulsory internship. The clinical rotations vary between 2 months and 2 weeks for clinical disciplines in the hospital, whereas 6 months are reserved for a rural/community posting. All training is from hospital-based specialists and public health experts. FPs or primary care doctors are not part of the faculty or training.

#### Postgraduate training

In South Africa, there has also been an option since 2007 to train as a FP through becoming a registrar in an accredited 4-year postgraduate programme. Postgraduate training aims to deliver an expert generalist who can work as a competent clinician throughout the district health system (including the district hospital), act as a consultant to the primary care team, be a mentor and capability builder, lead clinical governance, support a community-orientated primary care approach and provide clinical training to medical students, interns, clinical associates or registrars [20]. Although there are nine different training programmes, they are all constructed around a national set of learning outcomes, clinical skills list, portfolio of learning in the workplace, and a national exit examination.

In China, there is the possibility of full-time training over 3 years with 2.5 years in hospital rotations and then 6 months in the community. On-the-job training is offered for the specialists that have been converted to work as GPs before sitting the certification exams. Re-training for GPs to work in rural areas is also offered.

In Brazil, in order to be a FP, one must undergo a full-time 2-year residency programme. Most posts for family medicine residency training remain empty even with financial bonuses. Training takes place in the community (at least 10% of time), primary care facilities (at least 50% of time) and referral hospitals (at least 10% of time).

In India, the concept of family medicine training has been debated for several decades but has not been implemented at scale due to concerns about cost and the duration of training. A limited number of family medicine residency posts (200/year) have been introduced during the last decade. The current programme consists of 3 years full-time training, and out of the 3 years, 9 months are scheduled for community posting, while the rest of the training takes place in a hospital setting. Recent policy is supportive of training FPs for community health centres with an emphasis on surgical and anaesthetic skills. There have been other initiatives to up-skill the pool of existing primary care doctors through short courses that focus on key skills gaps such as anaesthesia, obstetrics and neonatal care.

South Africa, India and China have also introduced a mid-level doctor to strengthen district-level health services, who is trained in a 3-year degree programme. The intention is for them to strengthen services in rural and underserved areas as opposed to referral hospitals and urban areas. In China, mid-level doctors trained in this way can also become a full doctor by passing the national certification test following a 2-year residency. In India, there has been a long standing intention to introduce mid-level doctors, which is likely to be implemented soon with the plans to develop district hospitals as learning centres.

### Future directions

**Figure 1:**
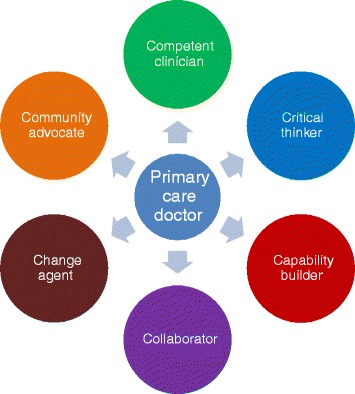
Roles of the South African primary care doctor.

In South Africa, strategies focus on both the training of primary care doctors and FPs. There is a process underway at the national level between all the medical schools to introduce a revised 2-year postgraduate diploma programme, which would re-orientate and up-skill the existing pool of primary care doctors for the future primary care system. This project has defined the roles of the future primary care doctor in South Africa as described below and shown in Figure 1 [21].

- Competent clinician: able to be clinically competent across the burden of disease and provide comprehensive patient-centred care
- Collaborator: able to work in a collaborative style as part of a multi-professional team
- Critical thinker: able to bring a degree of critical thinking to the team in terms of making sense of community data, health information or latest evidence and planning appropriate responses
- Change agent: able to actively contribute to the improvement of quality in the primary care services
- Capability builder: able to mentor, train or teach the other members of the primary care team where appropriate
- Community advocate: able to think about and advocate for the health needs of the local community served by the primary care team

At the same time, a national position paper on family medicine aims to train sufficient FPs for each district hospital, community health centre and sub-district as a short-term goal [20]. At the undergraduate level, plans are underway to create new medical schools, and at present, 1000 South Africans are being sent each year to train as doctors in Cuba.

In Brazil, the “More Doctors” Programme will allow foreign physicians to work at primary care health units without accreditation exams, for up to 3 years. They also plan to ensure that the number of residency vacancies is equivalent to the numbers of graduates from 2018. Admission to residency programmes for the general specialities (e.g. internal medicine, surgery) will require completion of the first year of the residency programme in family medicine. The Program for Professionals in Primary Health Care also offers incentives, such as no income tax, to doctors who work for at least 1 year in PHC. These policies should lead to a growth in the number of Brazilian doctors in PHC and training as FPs.

In India, there is a need to reach consensus on national policy and to implement this consistently across all states. There are plans to further develop the training of mid-level doctors, to allow nurse practitioners to practice independently, to train AYUSH practitioners in rural areas in prescribing of allopathic medicines, and to continue up-skilling primary care doctors. A recent report of India’s planning commission, the topmost policy-making body, estimated that India would require 15,000 FPs per year by 2030. However, it is not yet clear whether there will be widespread support for the training of FPs.

In China, the emphasis is on consolidating the new policy of strengthening primary care through the re-orientation of specialists to general practice and training of new general practitioners. There is a need to build trust amongst the public and expand the range and quality of the services in the new system.

### Reflections and discussion—on role and training of primary care doctor

#### Roles of the primary care doctor

Most countries have opted for a role of the doctor as part of a primary care team. In Brazil, every team must have a doctor, while in South Africa, the team is nurse-led but supported by a visiting doctor. In India, the doctor is one of several primary care providers, although the team is less clearly defined. Only China appears to have opted for a more doctor-led team with first-contact care from the doctor, which in some ways appears to be a reaction to poor quality primary care offered by less trained providers in the past.

Task-shifting to more available, less expensive and more retainable health workers is a clear strategy. The importance of a multi-professional primary care team which allows roles to be defined in a functional response to the needs of the community is emerging. This is in contrast to the way in which health professionals have traditionally defended their roles and identity in inter-professional rivalry that lacks alignment to the community’s needs or health system functioning.

The contribution of the doctor to the functioning of the whole team has implications for their roles, which goes beyond the obvious need for clinical competence. South Africa has recently defined these roles in some detail (Figure 1). In India, medical doctors working in PHC often complain that they are assigned administrative or managerial roles rather than being expected to work as clinicians. In China, because the primary care service is so underutilised, the clinical staff are often given public health tasks to complete, for example, to conduct HIV/sexually transmitted infections surveillance. In Brazil, FPs must also develop skills related to clinical governance; for example, they should know about the existing information systems in the national health system and how to analyse the data available, to assess health actions and to conduct health planning in their area.

All of these countries have recognised the importance of postgraduate training for family medicine or primary care, although the numbers of doctors with this training are small and insufficient to meet the needs. Brazil and China appear to have fully engaged with this issue in policy and planning with active strategies to attract, train and retain doctors in a career in primary care. South Africa appears to retain some ambivalence about FPs. On the one hand they are mentioned in policy, such as the national development plan, but on the other hand still struggle for registrar and FP posts. India appears to still be debating the issue and has not implemented its policy.

The underlying issue appears to be whether countries can afford to pay for more highly trained FPs and whether the extra cost would be worth it in terms of better health outcomes and a more functional district health care system. There are also concerns as to whether FPs are appropriately trained for the local community needs or whether training is based on imported curricula from high-resource settings. In South Africa, the government appears more convinced about the need for FPs at the district hospital than in primary care. There is a need for more evidence on the impact of FPs in low- and middle-income country settings.

One critical conceptual difference is the extent to which the doctor is orientated towards the community or the facility. Part of Brazil’s success appears to be its commitment to community-orientated primary care (COPC) at the heart of its PHC system, and South Africa is starting to introduce this approach. This changes the fundamental orientation of the system from being reactive, curative and facility-based to being proactive, preventative and community-based [22]. For example, in South Africa, the COPC approach has shown great promise in tackling the TB epidemic as this allows the identification of untreated TB patients and contacts, who would not be found in a facility-based approach [23]. China, however, appears committed to a more facility-based primary care system and has shied away from COPC as a reaction to the public’s lack of confidence in “barefoot doctors”.

In South Africa and India, the need for FPs to have an extended range of skills in anaesthesia, obstetrics and surgery is clear. This creates a tension between training FPs to offer these more procedural skills, for example, at the district hospital, where such skills are often missing, versus training them to be the front-line PHC workers as in Brazil and China.

In all these countries, the private sector does not reflect a PHC approach and often undermines it by offering direct access to specialist care that can be inappropriate and expensive.

#### Appropriate training for primary care

Going to scale with appropriately trained primary care doctors that are cost-effective appears to be the challenge faced by all these countries in strengthening PHC. The need for generalists to provide district hospital-type skills (e.g. anaesthesia, obstetrics and surgery) is also a feature of South Africa, China and India. A variety of strategies have been adopted in the short term to increase the number of primary care doctors, which include importing foreign (often Cuban) doctors, requiring all graduates to offer community service, requiring specialists to re-deploy as GPs or enlisting the help of other healing traditions (such as traditional Chinese herbalists or AYUSH practitioners).

Most countries have also introduced a variety of short courses and diplomas to re-orientate and up-skill the existing pool of primary care doctors (and in China even specialists) to serve the primary care system better.

Brazil appears to have fully embraced a model of postgraduate training for FPs at the policy level with the intention of filling its family health care teams with local and appropriately trained FPs. It is trying to go to scale and create incentives for doctors to choose this as a career pathway. In South Africa, there is still ambivalence amongst policy makers about the role of the FP, and we hope that experience with current FPs and the national position paper as well as ongoing research will help to establish consensus on the contribution of the FP and the need for a trained expert generalist as part of the system [24]. This also provides a more attractive career path for doctors in primary care. We need to increase the numbers of people training as FPs and enhance the capacity of the current system to support high-quality training and assessment in the national exit examination.

Despite the importance of PHC to health systems and communities, it is clear that primary care as a career choice lacks status for physicians. There may be many reasons for this including the lack of positive exposure in undergraduate education (this appears necessary to promote the career pathway but is not sufficient to ensure career choice), less remuneration and the need to work in more rural and remote areas. Countries are experimenting with ways of incentivising doctors to choose primary care as a career by, for example, ensuring exposure to primary care in undergraduate training, internship and as a requirement for residency training. Community service in South Africa may have had a paradoxical negative effect as newly qualified doctors often feel unsupported and overwhelmed. In China, PHC may be seen as simple activities for poor people and defined more by the gaps in services not covered by hospital-based care. Not needing to go to a generalist may be a kind of social status in some communities.

Training programmes need to identify the competencies required to address the local community’s health needs and priorities in the context of multi-professional teams, rather than replicating ideas and curriculum from more resource-rich contexts. We need to develop tools to evaluate primary care activities more clearly, such as the Primary Care Assessment Tool [24], integrate community-orientated thinking into primary care and teach an integrated comprehensive approach, rather than selected care, vertical programmes and biomedical approaches. Person-centred care needs to be at the heart of the training with competencies of generalism [25]. Inter-professional education should engage the role players early on in a collaborative complex adaptive approach to forming functional teams that meet the community’s needs [26].

Training and policy must aim for a high-quality primary care system. This will require conceptualising and changing the system as much as training appropriately for it. The experience of primary care should be “unexpectedly delightful” as one participant put it.

## Conclusion

Universal health coverage through primary health care is a laudable and important goal, but the quality of this primary health care needs to be such that the population has trust in the services and gains in health outcomes are clearly seen. Doctors with postgraduate training in family medicine or general practice are an essential component of ensuring this quality as part of a broader primary health care team. Countries need to fully embrace the cost of having such expert generalists at the forefront of their health care systems and to fully implement and support the global and national policy in this regard.

## Abbreviations

ASHA: Accredited Social Health Activist
AYUSH: Ayurveda, Yoga, Unani, Siddha, Homeopathy
BRICS: Brazil, Russia, India, China, South Africa
CHC: community health centre
COPC: community-orientated primary care
FP: family physician
GDP: gross domestic product
GP: general practitioner
HIV: human immunodeficiency virus
PHC: primary health care
TB: tuberculosis
WHR: World Health Report
